# A dual-function selection system enables positive selection of multigene CRISPR mutants and negative selection of *Cas9*-free progeny in *Arabidopsis*

**DOI:** 10.1007/s42994-023-00132-6

**Published:** 2024-01-22

**Authors:** Feng-Zhu Wang, Ying Bao, Zhenxiang Li, Xiangyu Xiong, Jian-Feng Li

**Affiliations:** State Key Laboratory of Biocontrol, Guangdong Provincial Key Laboratory of Plant Resources, School of Life Sciences, Sun Yat-sen University, Guangzhou, 510275 China

**Keywords:** CRISPR, Genome editing, d-amino acid oxidase, *Cas9*-free, Selection marker

## Abstract

The CRISPR/Cas9 technology revolutionizes targeted gene knockout in diverse organisms including plants. However, screening edited alleles, particularly those with multiplex editing, from herbicide- or antibiotic-resistant transgenic plants and segregating out the *Cas9* transgene represent two laborious processes. Current solutions to facilitate these processes rely on different selection markers. Here, by taking advantage of the opposite functions of a d-amino acid oxidase (DAO) in detoxifying d-serine and in metabolizing non-toxic d-valine to a cytotoxic product, we develop a DAO-based selection system that simultaneously enables the enrichment of multigene edited alleles and elimination of *Cas9*-containing progeny in *Arabidopsis thaliana*. Among five DAOs tested in *Escherichia coli*, the one encoded by *Trigonopsis variabilis* (TvDAO) could confer slightly stronger d-serine resistance than other homologs. Transgenic expression of *TvDAO* in *Arabidopsis* allowed a clear distinction between transgenic and non-transgenic plants in both d-serine-conditioned positive selection and d-valine-conditioned negative selection. As a proof of concept, we combined CRISPR-induced single-strand annealing repair of a dead *TvDAO* with d-serine-based positive selection to help identify transgenic plants with multiplex editing, where d-serine-resistant plants exhibited considerably higher co-editing frequencies at three endogenous target genes than those selected by hygromycin. Subsequently, d-valine-based negative selection successfully removed *Cas9* and *TvDAO* transgenes from the survival offspring carrying inherited mutations. Collectively, this work provides a novel strategy to ease CRISPR mutant identification and *Cas9* transgene elimination using a single selection marker, which promises more efficient and simplified multiplex CRISPR editing in plants.

**Supplementary Information:**

The online version contains supplementary material available at 10.1007/s42994-023-00132-6.

## Introduction

CRISPR/Cas9 is a revolutionary genome editing technology inspired by the adaptive immune system of bacteria and archaea for defending against viral infection and has been widely applied to gene knockout in various organisms including plants (Gürel et al. 2020). Current CRISPR/Cas9 applications in plants predominantly rely on *Agrobacterium tumefaciens*-mediated genetic transformation, by which the T-DNA encoding genome editing reagents are stably integrated into the plant genome. In the model species *Arabidopsis thaliana*, *Cas9* overexpression by a strong constitutive promoter (e.g., *35S* promoter) often results in chimeric mutations in the T_1_ generation (Feng et al. 2014; Tsutsui and Higashiyama 2017; Wolabu et al. 2020). In contrast, using an egg cell-specific promoter to drive *Cas9* expression can produce nonchimeric (that is, heterozygous, homozygous or biallelic) T_1_ mutants, although with a relatively lower efficiency (Wang et al. 2015). The identification of CRISPR-edited transgenic plants routinely requires PCR-based genotyping and/or target amplicon sequencing, which is time-consuming and costly. Therefore, strategies to enrich CRISPR-edited plants during mutant screening are highly desirable, particularly in the case of multiplex editing. Recently, surrogate systems based on the restoration of a defective hygromycin resistance gene (*HygR*) have been developed to confer effective enrichment of rice mutant plants in CRISPR editing (Tian et al. 2023), base editing (Xu et al. 2020a), and prime editing (Xu et al. 2020b). CRISPR-mediated disruption of *MAR1*, an endogenous transporter gene, has been demonstrated to facilitate co-selection of target gene editing in *Arabidopsis* and tomato (Rinne et al. 2021). A visible selection system has also been established in *Arabidopsis* to enrich CRISPR-induced mutants in the T_1_ generation, which is based on a glabrous phenotype caused by mutating the *GLABRA2* gene that is involved in trichome formation (Kong et al. 2021).

On the other hand, the retention of *Cas9* transgene after target gene knockout can significantly increase the risk of off-target editing (Fu et al. 2013; Zhang et al. 2018; Cullot et al. 2019). Moreover, in transgenic complementation experiments for validating the mutant phenotype and target gene correlation, it is necessary to eliminate the *Cas9* before the target gene is re-introduced into the edited mutant plant. Furthermore, in CRISPR-mediated molecular breeding, the inclusion of the *Cas9* transgene in crops raises regulatory concerns. Although the *Cas9* transgene can be eliminated through genetic segregation, the process of screening non-transgenic progeny with desired mutations is laborious. Therefore, several strategies have been developed to facilitate the isolation of *Cas9*-free mutant plants (He and Zhao 2019). One approach for eliminating *Cas9* from genome-edited plants involves RNA interference-induced herbicide susceptibility, which allows the survival of *Cas9*-free plants (Lu et al. 2017). Alternatively, non-transgenic seeds or plants could be visually distinguished from transgenic ones by integrating a transgene giving rise to detectable fluorescence (Gao et al. 2016; Wang and Chen 2020), early flowering (Liu et al. 2019; Lao et al. 2023), or anthocyanin accumulation (Liu et al. 2019). Although He and colleagues have created an ingenious strategy, called TKC (Transgene Killer CRISPR), enabling self-elimination of the CRISPR construct after targeted gene editing in rice (He et al. 2018), this approach cannot be applied to *Arabidopsis* that is transformed by the floral dip method (Clough and Bent 1998) rather than tissue culture. Of note, to date, no strategy has been developed to enable both the enrichment of CRISPR-edited mutants and subsequent elimination of the *Cas9* transgene from the mutant offspring.

d-amino acid oxidases (DAOs) are specialized enzymes for d-amino acid metabolism, which are encoded by many eukaryotes but not plants (Pilone 2000). Interestingly, d-serine at high concentrations is toxic to plants but can be detoxified by transgenically expressed DAOs (Erikson et al. 2004; Lim et al. 2007; Gisby et al. 2012). In contrast, d-valine is non-toxic to plants but can be metabolized to a toxic product by a *DAO* transgene (Erikson et al. 2004; Gisby et al. 2012). In this study, we leveraged the dual functions of a DAO in metabolizing different d-amino acid substrates to develop a surrogate selection system for simplifying genome editing in *Arabidopsis*. This system can simultaneously facilitate the screening of CRISPR-edited alleles and subsequent elimination of mutant progeny containing the *Cas9* transgene.

## Results

### ***TvDAO*** confers d-serine resistance in ***Escherichia coli***

**Fig. 1:**
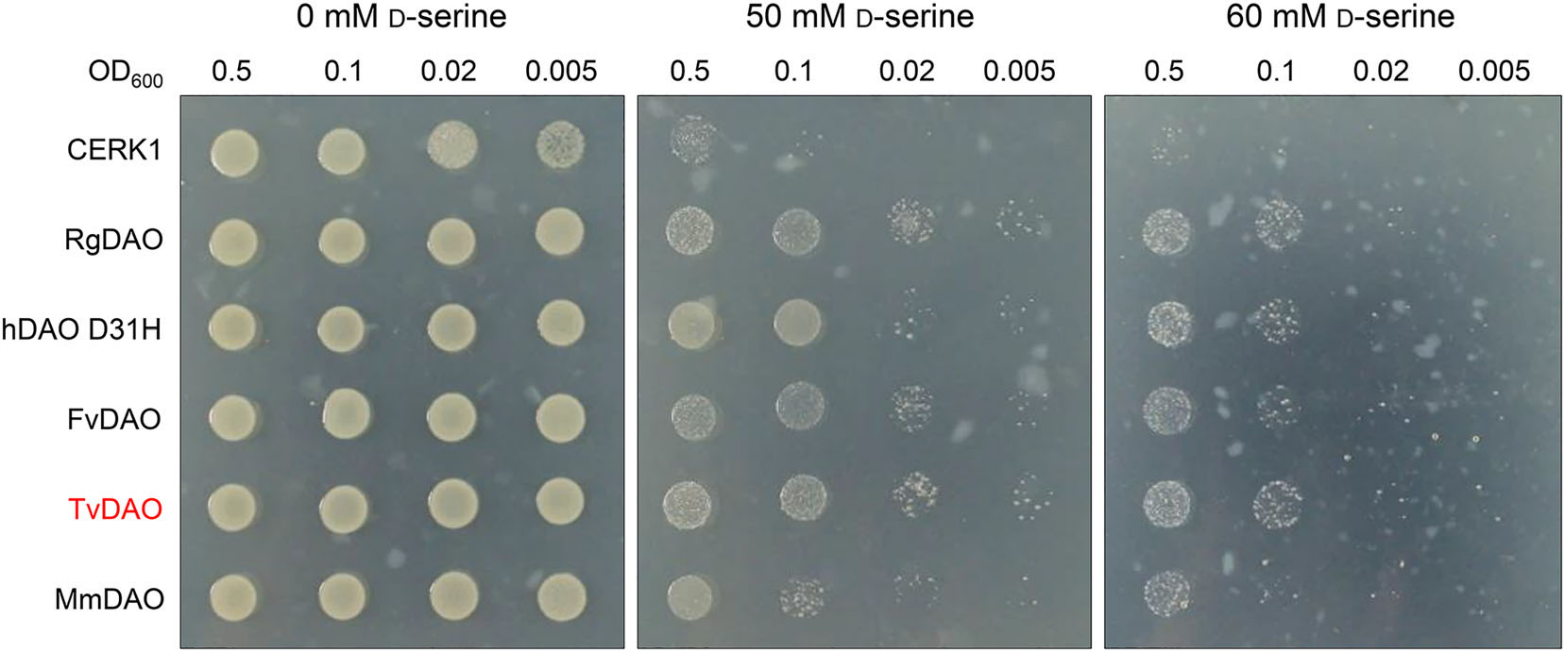
d-amino acid oxidase confers d-serine tolerance in *Escherichia coli*. *E. coli* cells expressing *Rhodotorula gracilis d*-*amino acid oxidase* (*RgDAO*), *Homo sapiens DAO* (*hDAO D31H*), *Fusarium verticillioides DAO* (*FvDAO*), *Trigonopsis variabilis DAO* (*TvDAO*), or *Mus musculus DAO* (*MmDAO*) were diluted to indicated cell concentrations (OD_600_) and inoculated on the growth medium containing 0, 50 or 60 mM d-serine. *E. coli* cells expressing *Arabidopsis thaliana CERK1* encoding a receptor kinase were used as a negative control. TvDAO used for further study is highlighted in red. These experiments were repeated three times with similar results

To utilize a DAO-based surrogate selection marker for CRISPR editing, we first sought to screen a DAO with robust catalytic activity in detoxifying d-serine. For this purpose, we selected five homologous *DAO*s (Pilone 2000; Pollegioni et al. 2007, 2018) from *Rhodotorula gracilis* (*RgDAO*), *Homo sapiens* (*hDAO D31H*), *Fusarium verticillioides* (*FvDAO*), *Trigonopsis variabilis* (*TvDAO*), and *Mus musculus* (*MmDAO*), respectively. These *DAO*s were codon-optimized (Supplementary Sequences) for optimal expression in *Arabidopsis* before gene synthesis. When using the *Arabidopsis* protoplast-based transient assay to evaluate the activity of these DAOs in detoxifying d-serine, we observed that *Arabidopsis* protoplasts were not sensitive to d-serine treatment. Considering that *E. coli* is a frequently used heterologous system for evaluating the function of a DAO in detoxifying d-amino acids (Pollegioni et al. 2007), we switched to *E. coli* to compare the activities of these DAOs. In three biological replicates, *E. coli* cells expressing *TvDAO* exhibited slightly better growth on the d-serine-containing medium than those expressing the other DAOs, particularly at a d-serine concentration of 60 mM (Fig. 1). Conversely, *E. coli* cells expressing the negative control *CERK1*, which encodes a receptor-like kinase from *Arabidopsis*, suffered obvious growth arrest in the presence of d-serine (Fig. 1). These findings suggest that all *DAO* transgenes are able to confer d-serine tolerance in *E. coli*.

### *TvDAO* enables both positive and negative selection for transgenic *Arabidopsis* plants

**Fig. 2:**
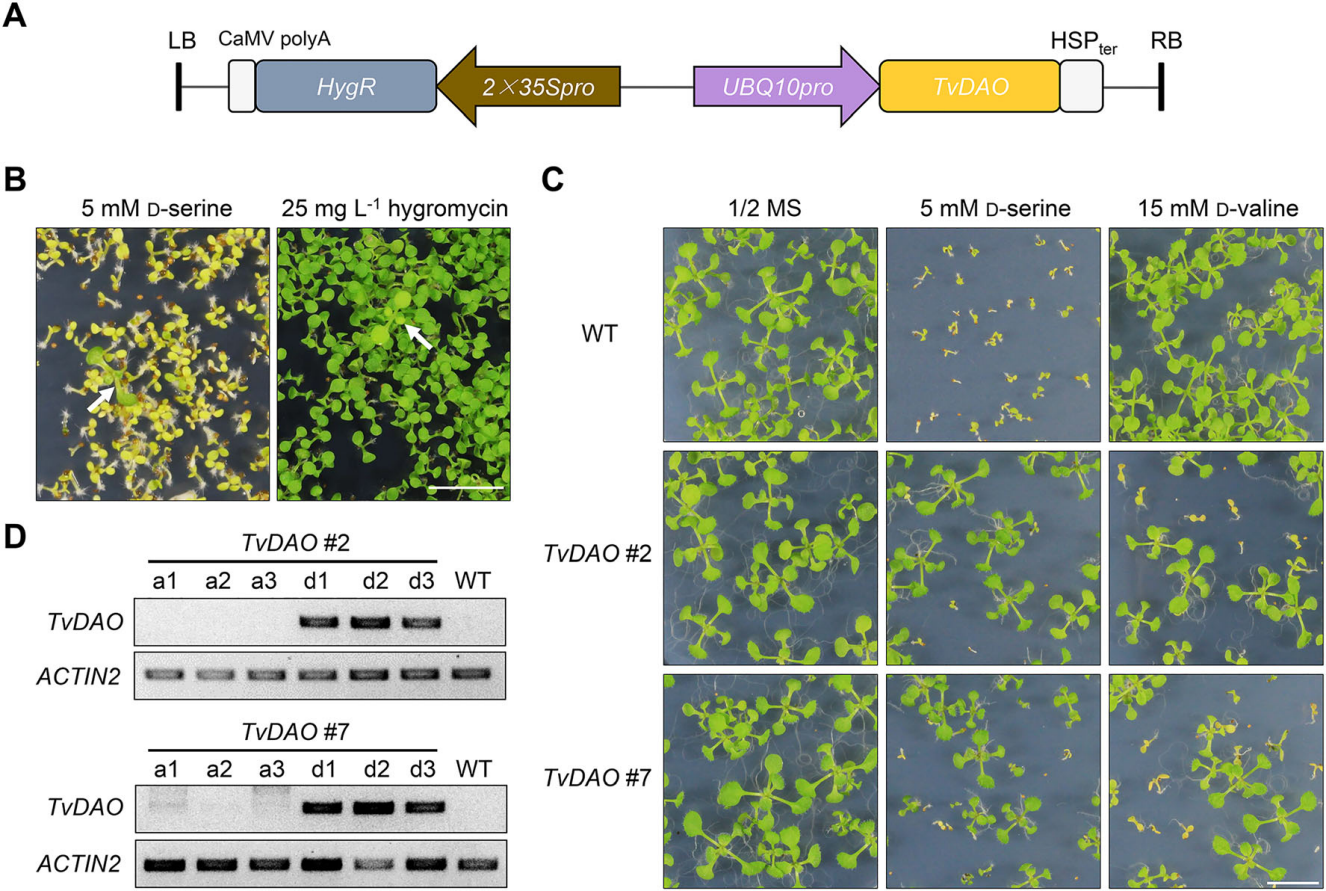
*TvDAO* enables both positive and negative selection of transgenic *Arabidopsis* plants. **A** Diagram of the expression cassettes for *TvDAO* and hygromycin resistance gene (*HygR*) in the binary plasmid. LB, T-DNA left border; RB, T-DNA right border; HSP_ter_, Heat shock protein terminator. **B** Transgenic *TvDAO* plant selected by d-serine shows a clearer resistance phenotype than that selected by hygromycin. Seedlings indicated by white arrows are transgenic plants. Scale bar = 1 cm. **C** Progeny of d-serine-resistant lines, *TvDAO* #2 and *TvDAO* #7, show resistance segregation in both d-serine-based positive selection and d-valine-based negative selection. Scale bar = 1 cm. **D** PCR-based genotyping reveals that d-valine resistance or susceptibility is consistent with the absence or presence of *TvDAO* transgene. a1-a3 and d1-d3 are randomly selected alive or dying seedlings, respectively, on the d-valine-containing medium. *ACTIN2* was used as an internal control

Considering that the TvDAO is not only smaller in size but also exhibited modestly stronger activity in conferring d-serine resistance in *E. coli* (Fig. 1), we next focused on *TvDAO* and attempted to validate whether it could be employed for d-serine-based positive selection and d-valine-based negative selection of transgenic plants. To this end, we generated transgenic *Arabidopsis* plants expressing *TvDAO* along with the *HygR* gene (Fig. 2A) for side-by-side comparison. When screening the transgenic T_1_ plants on 1/2 Murashige and Skoog (MS) medium containing 5 mM d-serine or 25 mg L^−1^ hygromycin, the transgenic plants selected by d-serine exhibited a clearer resistance phenotype than those selected by hygromycin (Fig. 2B), implying that *TvDAO* may be a better selection marker gene than *HygR* in *Arabidopsis*.

The T_2_ generation of two representative lines, *TvDAO* #2 and *TvDAO* #7, showed resistance segregation when germinated on the medium containing d-serine or d-valine, whereas all wild-type plants were sensitive to d-serine but grew well on the d-valine-containing medium (Fig. 2C). Consistently, PCR-based genotyping of three randomly selected healthy seedlings (i.e., a1-a3) or dying seedlings (i.e., d1-d3) from the progeny of *TvDAO* #2 or *TvDAO* #7 grown in the presence of d-valine showed that the a1-a3 were transgene-free, while the d1-d3 were transgenic (Fig. 2D). These results indicated that the *TvDAO*-based reporter supports both positive selection of transgenic plants by d-serine and negative selection of transgene-free plants by d-valine.

### *TvDAO*-based selection system facilitates the screening of CRISPR-edited alleles in *Arabidopsis*

**Fig. 3:**
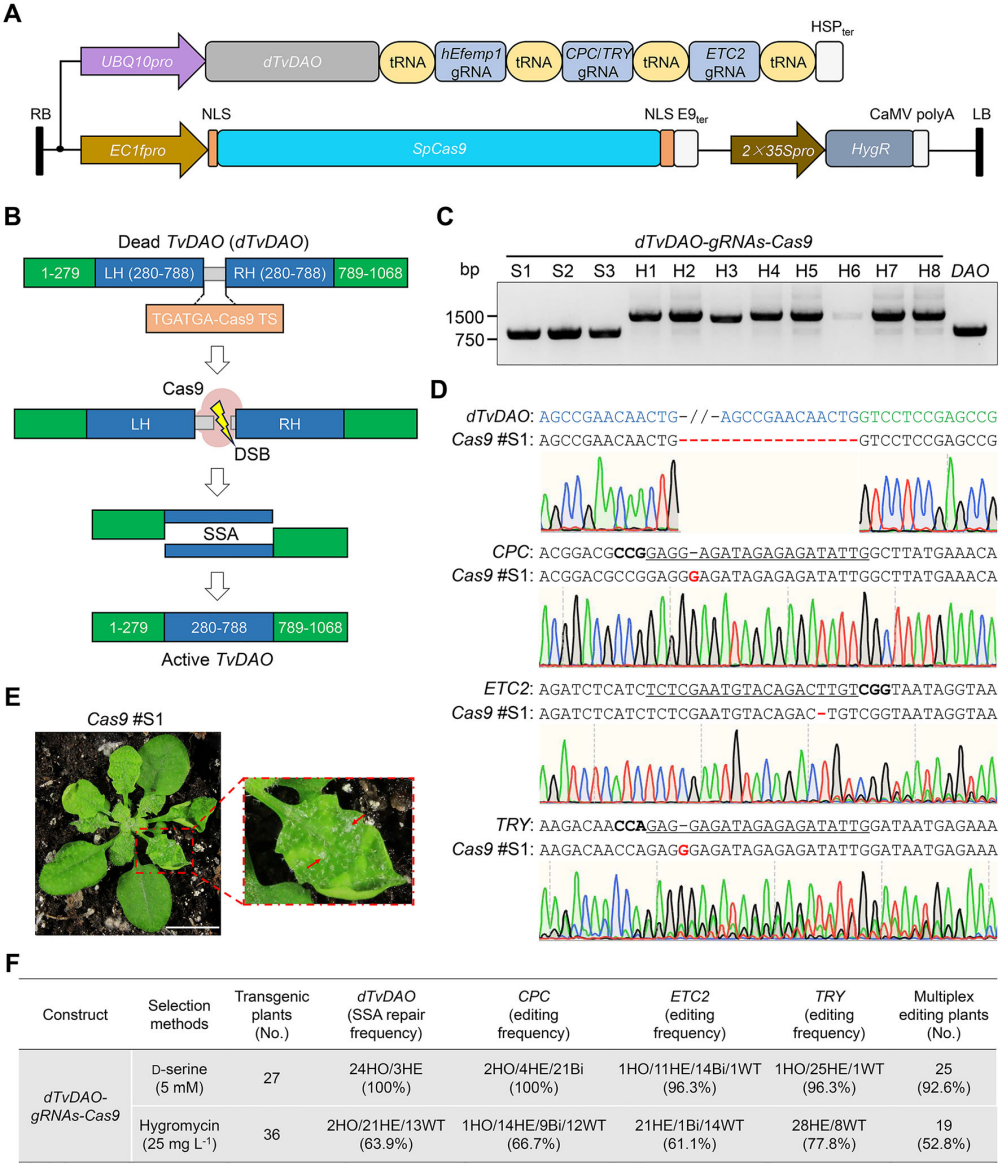
*TvDAO* helps to enrich CRISPR-edited alleles in *Arabidopsis*.** A** Diagram of the expression cassettes for d*TvDAO*, *Cas9* (*SpCas9*), gRNAs and hygromycin resistance gene (*HygR*) in the binary plasmid. NLS, nuclear localization signal; E9_ter_, E9 terminator. **B** Rationale of CRISPR-induced single-strand annealing (SSA) repair to restore a functional *TvDAO* selection marker. In the dead *TvDAO* (*dTvDAO)* reporter, the most 5′ (1–279) and 3′ segments (789–1068) of *TvDAO* were colored in green. The middle segment (280–788) of *TvDAO* was colored in blue and the two copies (LH and RH) of it were spacered by two consecutive stop codons and a Cas9 target site from the human *hEfemp1* locus. **C** PCR-based genotyping confirms successful SSA repair of *dTvDAO* in d-serine-resistant transgenic T_1_ plants. S, d-serine selection. H, hygromycin selection. Transgenic *TvDAO* #2 plant was used as a positive control. **D** Sanger sequencing of target amplicons validates the editing of all four target sites (*dTvDAO*, *CPC*, *ETC2* and *TRY*) in the *dTvDAO-gRNAs-Cas9* #S1 (*Cas9* #S1) plant. Blue letters in *dTvDAO* represent the C-terminal coding sequences of LH and RH, while green letters represent the coding sequences adjacent to the C-terminus of RH. Black bold letters mark PAMs and target sequences of gRNAs are underlined. Deletions are indicated by red dashes, while insertions are colored in red. **E** The *Cas9* #S1 plant exhibits visible clustering of trichomes on the leaf surface as indicated by red arrows. Scale bar = 1 cm. **F** Summary of mutation frequencies at individual target sites in transgenic T_1_ plants selected by d-serine or hygromycin. HO, HE, Bi and WT denote homozygote, heterozygote, bi-allele and wild type, respectively

Given that *TvDAO* can be used for both positive and negative selection in *Arabidopsis*, depending on the d-amino acid substrate, we hypothesized that it would serve as an excellent surrogate selection marker for enriching CRISPR-edited alleles and then eliminating *Cas9*-containing progeny. To achieve this, CRISPR-induced single-strand annealing (SSA) repair converting a dead *TvDAO* (*dTvDAO*) reporter to an active form was devised (Fig. 3A, and B). SSA is a homologous recombination-based DNA repair mechanism that promotes recombination between two repetitive DNA sequences flanking a double-stranded break (DSB), resulting in the deletion of the intervening fragment (Endo et al. 2021; Vu et al. 2022). To validate the effectiveness of SSA repair in plant cells, we tested a dead *GFP* (*dGFP*) reporter (Fig. S1A), in which *GFP* was split into two fragments sharing a 321-bp overlapping sequence (nucleotides 199–519). These two fragments were separated by a linker containing two consecutive stop codons and a human genome-derived Cas9 target site (TGATGA-Cas9 TS). To optimize the CRISPR-induced SSA efficiency, we compared three different human target sites (i.e., *hEMX1-1*, *hEMX1-2* and *hEfemp1*) in the *dGFP* reporter using corresponding gRNAs, namely *hEMX1*-gRNA1, *hEMX1*-gRNA2 (Cong et al. 2013), and *hEfemp1*-gRNA (Xu et al. 2020a). None of these gRNAs have predictable off-target sites in *Arabidopsis*. Transfection of the *dGFP* reporters alone into *Arabidopsis* protoplasts failed to produce any GFP fluorescence, whereas co-expressing *Cas9* and a gRNA targeting the cognate human target site efficiently restored *GFP* expression (Fig. S1B–D). Considering comparable *GFP* regeneration efficiencies between the three human target site/gRNA pairs, we chose the *hEfemp1* TS/*hEfemp1*-gRNA pair to build the *dTvDAO* reporter because this pair has been used *in planta* in a recent study (Xu et al. 2020a).

We designed the *dTvDAO* reporter by duplicating nucleotides 280–788 of the *TvDAO* coding sequence and inserting the linker of TGATGA-*hEfemp1* TS between the two duplicated sequences (i.e., LH and RH) (Fig. 3B). We constructed the binary plasmid *dTvDAO-gRNAs-Cas9* [*pHEE401E-UBQ10pro::dTvDAO-poly A-(tRNA- gRNA)s*] to express *Cas9* using the egg cell-specific EC1.2-EC1.1 fusion (EC1f) promoter (Wang et al. 2015) and to express *dTvDAO* and polycistronic tRNA-gRNA repeats as a single transcript unit using the *UBQ10* promoter (Tang et al. 2019) (Fig. 3A). The polycistronic tRNA-gRNA repeats could produce multiple gRNAs through the endogenous tRNA-processing machinery (Xie et al. 2015). In addition to the *hEfemp1*-gRNA, we incorporated two additional gRNAs in the tRNA-gRNA repeats that target to three endogenous genes related to trichome development in *Arabidopsis*, namely *ETC2*, *CPC*, and *TRY* (Kirik et al. 2004). *ETC2* was targeted by one gRNA (i.e., *ETC2*-gRNA), whereas *CPC* and *TRY* were targeted by the other gRNA (i.e., *CPC*/*TRY*-gRNA). Notably, the *pHEE401E* vector also encodes the *HygR* (Fig. 3A), allowing hygromycin selection of transgenic plants as an alternative option. As a negative control, we constructed the binary plasmid *dTvDAO-Cas9* (*pHEE401E-UBQ10pro::dTvDAO-poly A*) without polycistronic tRNA-gRNA repeats. None of the transgenic T_1_ plants expressing *dTvDAO-Cas9* survived d-serine selection, validating that the *dTvDAO* reporter, by itself, cannot encode an active DAO.

We harvested a total of 3.3 mL seeds from the plants transformed using the *Agrobacterium* cells carrying the binary plasmid *dTvDAO-gRNAs-Cas9*. These seeds were divided into two fractions, one (~ 3 mL seeds) for d-serine selection and the other (~ 0.3 mL seeds) for hygromycin selection. Among a total of 28 d-serine-resistant plants obtained, 27 (that is, S1-S28 except S15) were identified by PCR-based genotyping as transgenic plants, which all generated *TvDAO* amplicons with an identical size as those from the *TvDAO* transgenic line #2 (Figs. 3C and S2). This indicated successful recovery of *TvDAO* by SSA, which was further confirmed by Sanger sequencing of *TvDAO* amplicons from these plants (Fig. 3D). Among a total of 40 hygromycin-resistant plants obtained, 36 (that is, H1-H40 except H9, H14, H15 and H23) were identified as transgenic plants, which produced PCR amplicons of edited or non-edited *dTvDAO* (Figs. 3C and S2).

All 27 d-serine-resistant transgenic T_1_ plants harbored mutations for at least two of the three endogenous target genes (Fig. S3). Strikingly, 25 out of the 27 (92.6%) contained concurrent mutations across all three genes (Figs. 3D and S3). Consistently, slightly clustered trichomes could be seen on the leaf surface of some plants (Fig. 3E), as exemplified by *Cas9* #S1 that harbored homozygous mutation in *CPC* and heterozygous mutations in *ETC2* and *TRY* (Figs. 3D and S3). By contrast, only 19 out of the 36 (52.8%) hygromycin-resistant transgenic T_1_ plants contained concurrent mutations for all three target genes (Fig. S3). Notably, there were five plants (that is, H1, H27 and H33-35) containing no mutations at all for the four target loci including *dTvDAO* (Figs. S3 and S4). Importantly, d-serine-resistant transgenic plants not only showed an exceedingly higher co-editing frequency but also higher editing frequencies at individual endogenous target sites than hygromycin-resistant transgenic plants, namely 100% versus 66.7% for *CPC*, 96.3% versus 61.1% for *ETC2*, and 96.3% versus 77.8% for *TRY* (Fig. 3F). These findings demonstrated that the *dTvDAO* surrogate selection marker can ease the screening of mutant alleles produced by multiplex CRISPR editing in *Arabidopsis*.

### *TvDAO*-based selection system facilitates the identification of *Cas9*-free progeny

**Fig. 4:**
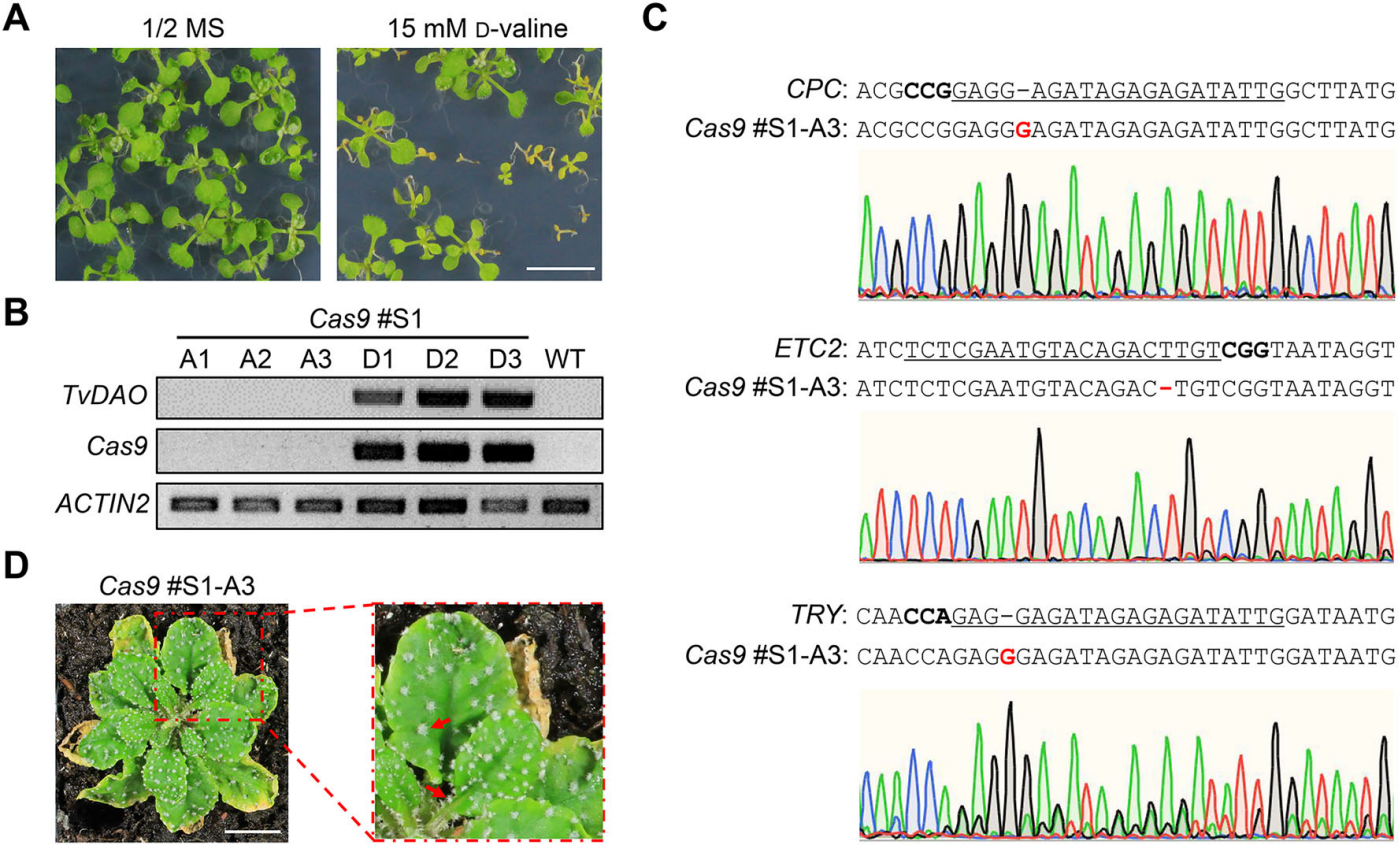
*TvDAO* can be used to eliminate *Cas9*-carrying mutant progeny.** A** Progeny of the transgenic *Cas9* #S1 plant exhibit resistance segregation on 1/2 MS medium containing 15 mM d-valine. Scale bar = 1 cm. **B** PCR-based genotyping confirms transgene elimination in survival T_2_ plants of *Cas9* #S1 under d-valine-conditioned negative selection. A1-A3 and D1-D3 are randomly selected alive or dying seedlings, respectively. **C** Sanger sequencing of target amplicons validates inherited mutations at the three endogenous target sites in the T_2_ plant *Cas9* #S1-A3. Black bold letters mark PAMs and target sequences of gRNAs are underlined. Insertion and deletions are in red. **D** The *Cas9* #S1-A3 plant displays highly clustered trichomes as indicated by red arrows. Scale bar = 1 cm

Next, we evaluated whether the *TvDAO*-based selection system could also be used for identifying *Cas9*-free mutant progeny. To this end, we germinated the T_2_ progeny of the *Cas9* #S1 plant on the 1/2 MS medium, with or without 15 mM d-valine. As anticipated, some seedlings were sensitive to d-valine (Fig. 4A). PCR-based genotyping revealed that three randomly selected healthy seedlings (i.e., A1-A3) were all *Cas9-* and *TvDAO*-free, whereas three randomly selected dying seedlings (i.e., D1-D3) all contained the transgenes (Fig. 4B). Sanger sequencing of target amplicons validated stable inheritance of T_1_ mutations at all three endogenous target sites in the T_2_ plant *Cas9* #S1-A3, in which the mutations at the *ETC2* and *TRY* loci became homozygous (Fig. 4C). Therefore, the *Cas9* #S1-A3 plant represented a *Cas9*-free *cpc etc2 try* triple mutant allele, which displayed highly clustered trichomes (Fig. 4D) as previously reported (Kirik et al. 2004). These results suggested that d-valine-based negative selection allows efficient identification of *Cas9*-free mutant alleles.

## Discussion

**Fig. 5:**
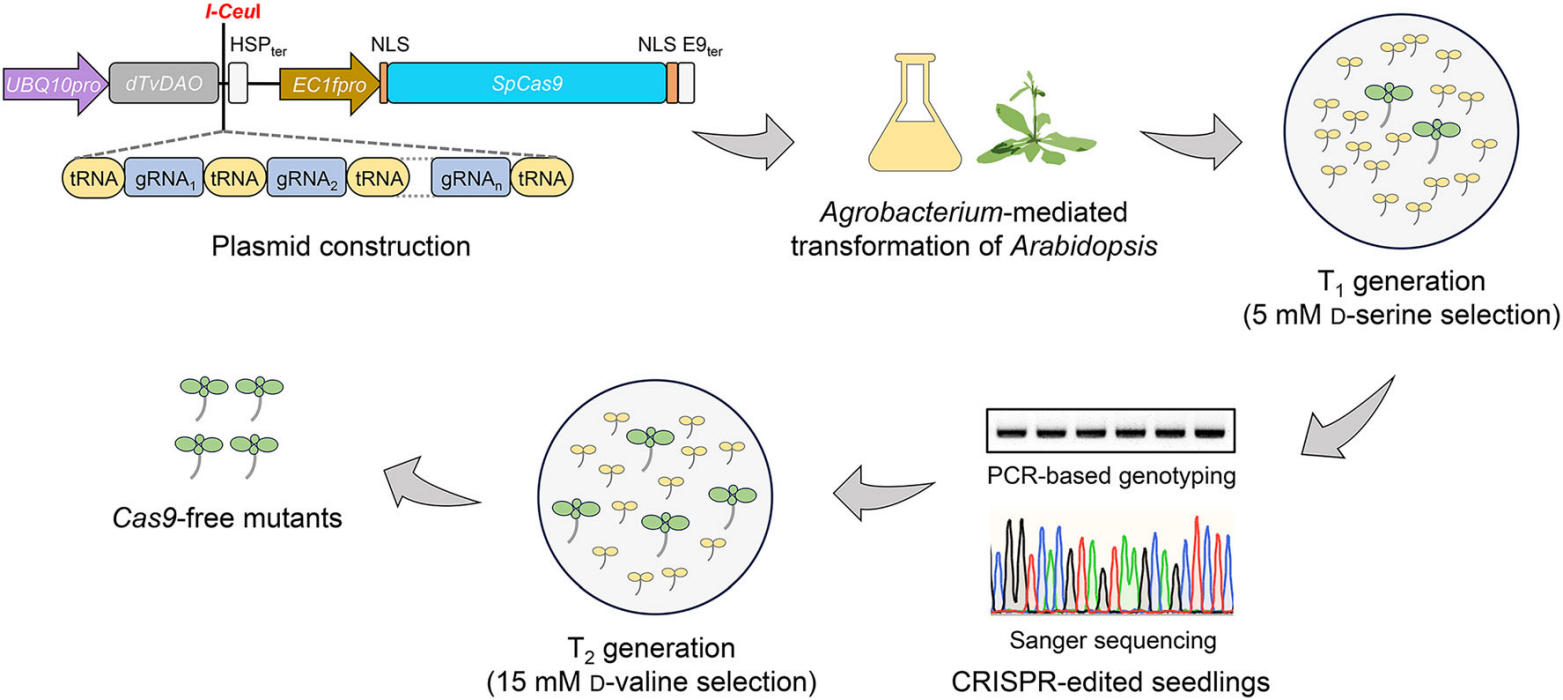
Overview of *TvDAO*-facilitated screening for CRISPR-edited and *Cas9*-free *Arabidopsis* mutant alleles. The gRNAs targeting the human *hEfemp1* site in *dTvDAO* (gRNA_1_), or endogenous genes of interest (gRNA_2_ to gRNA_n_), are expressed as polycistronic tRNA-gRNA repeats, which are assembled by Golden Gate assembly and inserted into the *pHEE401E* vector, via the rare-cut restriction site *I-Ceu*I. This allows gRNAs to be produced along with the *dTvDAO* reporter as a single transcript unit driven by the strong constitutive *UBQ10* promoter. *Cas9* is expressed under the egg cell-specific *EC1f* promoter. By *Agrobacterium*-mediated transformation, transgenic *Arabidopsis* plants expressing dTvDAO, gRNAs, and Cas9 are generated. T_0_ seeds are selected by 5 mM d-serine to enrich CRISPR-edited T_1_ alleles (green alive seedlings), which are further validated by target amplicon Sanger sequencing. Subsequently, their *Cas9*-free T_2_ alleles (green healthy seedlings) are selected by 15 mM d-valine

Screening Cas9-induced mutant alleles from herbicide- or antibiotic-resistant transgenic plants and isolating *Cas9*-free mutant progeny are two labor-consuming processes during plant genome editing. Although several surrogate selection systems have been explored to facilitate either process (Liu et al. 2019; Wang and Chen 2020; Xu et al. 2020a, 2020b; Kong et al. 2021; Rinne et al. 2021; Lao et al. 2023; Tian et al. 2023), to our knowledge, no selection marker can work for both processes. In this work, we demonstrate an innovative approach of using a *TvDAO*-based reporter as a single surrogate selection marker to ease the two demanding processes in *Arabidopsis*, particularly during multiplex CRISPR editing. Our streamlined approach involves five steps (Fig. 5): (1) construction of the *dTvDAO* reporter-containing binary plasmid co-expressing Cas9/gRNAs, in which multiple gRNAs are assembled as polycistronic tRNA-gRNA repeats; (2) *Agrobacterium*-mediated transformation of *Arabidopsis*; (3) selection of T_1_ generation using 5 mM d-serine; (4) Sanger sequencing of target amplicons to validate multigene editing in survival T_1_ alleles; (5) selection of T_2_ generation using 15 mM d-valine to obtain survival *Cas9*-free mutants.

In this study, we leveraged the CRISPR-induced SSA repair for seamless restoration of a reporter gene from its inactive version. Indeed, this strategy worked as expected in both *Arabidopsis* protoplasts and transgenic plants, for both *GFP* and *TvDAO* (Figs. 3 and S1). Notably, when we combined the CRISPR-induced SSA repair of the non-functional *dTvDAO* with d-serine-conditioned positive selection, we could observe significantly increased multiplex editing at the three endogenous target sites when compared to the routinely used hygromycin selection (Figs. 3F and S3), suggesting that multiple co-expressed gRNAs can mediate CRISPR editing with a considerable level of co-efficiency. Moreover, we noted an obvious trend that d-serine selection greatly stimulated *TvDAO* editing, as 24 out of the 27 (88.9%) d-serine-resistant T_1_ plants carried homozygous *TvDAO* mutations (Fig. S3). In contrast, only 2 out of the 36 (5.6%) hygromycin-resistant T_1_ plants contained homozygous *TvDAO* mutations (Fig. S3). It is very likely that the selection pressure, per se, also promotes the activity of CRISPR/Cas9 to produce functional *TvDAO* for plant survival. These combinatorial effects thus allow our surrogate selection system to effectively enrich mutant alleles with multigene editing.

It has not escaped our attention that, when transformed with the same binary construct conferring both d-serine and hygromycin resistance, transgenic plants selected by d-serine exhibited a reduced positive rate relative to those selected by hygromycin (Fig. 3E). A reasonable explanation for this observation is that the SSA is only a minor repair mechanism for DNA DSBs in higher eukaryotes (Stinson and Loparo 2021; Oh and Myung 2022), thus being outcompeted by the dominant non-homologous end joining (NHEJ) repair system (Fiorenza et al. 2001). Therefore, some transgenic plants with a Cas9-induced DSB in the *dTvDAO* reporter failed to generate an active *TvDAO*, via the SSA repair, and were killed by d-serine selection. The low efficiency of SSA repair in regenerating *TvDAO* is thus a bottleneck in our current strategy. One way for future optimization could be to use the NHEJ-based traffic light reporter (de Jong et al. 2020) of *dTvDAO* to replace the SSA-based *dTvDAO* reporter. In the so-called traffic light reporter, a spacer would be inserted after the start codon of *TvDAO*, which contains a Cas9 target sequence (such as the *hEfemp1* TS) and a downstream stop codon that is in frame with the start codon. When Cas9 induces a DSB at the spacer, the NHEJ-mediated frameshift of either + 1 nt or + 2 nt in this spacer would dismiss the stop codon and restore TvDAO production.

In summary, our TvDAO-based selection system allows both d-serine-based positive selection to facilitate CRISPR mutant screening and subsequent d-valine-based negative selection to remove Cas9-containing mutant offspring, thus significantly improving the time and labor efficiencies in isolating *Cas9*-free multigene mutant alleles in *Arabidopsis*. Given that d-serine is toxic for a wide range of plant species including tobacco, tomato, spruce, poplar, maize, and barley (Erikson et al. 2004) and that the DAO-induced d-serine resistance has also been validated in tobacco (Lim et al. 2007; Gisby et al. 2012), it is possible to transfer our *TvDAO*-based selection system to other plant species to simplify multiplex CRISPR editing. Meanwhile, a modified *dTvDAO* selection system should be useful in promoting base editing or prime editing in plants.

## Materials and methods

### Plant materials and growth conditions

The *Arabidopsis thaliana* ecotype Col-0 was used as the wild-type plants in this study. The transgenic T_1_ plants were screened on 1/2 × MS medium containing 5 mM d-serine or 25 mg L^−1^ hygromycin. The T_2_ plants were screened on 1/2 MS medium containing 15 mM d-valine. After stratification at 4 °C for 2 days, the seeds were grown in a plant growth chamber (Ningbo Saifu, China) under a photoperiod of 12 h light (75 μmol m^−2^ s^−1^) at 23 °C and 12 h dark at 21 °C. The resistant seedlings were transferred to Jiffy soil (Jiffy Group, Netherlands) and grown in a plant growth room under a photoperiod of 12 h light at 23 °C and 12 h dark at 21 °C, with humidity maintained at 65%.

### Plasmid construction

The coding sequences of *RgDAO*, *hDAO D31H*, *FvDAO*, *TvDAO*, and *MmDAO* were codon-optimized according to the codon bias of *Arabidopsis thaliana* (Supplemental sequences). The sequences were then synthesized and inserted into the *BamH*I and *Stu*I sites of the HBT-35SPPDK-2 × FLAG vector by GENCEFE Biotech Company (Wuxi, China). For CRISPR-induced SSA repair of *dGFP*, *dGFP* containing two overlapping fragments (nucleotides 199–519) of *GFP* and TGATGA-Cas9 TS was cloned by overlap PCR, then inserted into the *BamH*I and *Stu*I sites of the HBT-35SPPDK-2 × HA vector, resulting in the *HBT-35SPPDK-dGFP(TS)-2* × *HA* plasmid. The AtU6-26pro::gRNA expression cassettes were assembled by oligo phosphorylation and annealing, and then inserted into the *Bsa*I site of the pUC119 vector.

To generate the *pYL-UBQ10pro::TvDAO-2* × *HA-poly A-HSP*_*ter*_ (*TvDAO*) plasmid, the UBQ10 promoter, *TvDAO*, 2 × HA, and HSP terminator were cloned, assembled by overlapping extension PCR, then inserted into the *BamH*I and *Nco*I sites of the pYL vector using the ClonExpress MultiS One Step Cloning Kit (Vazyme, China). Similarly, the coding sequence of *UBQ10pro::dTvDAO-2* × *HA-ployA-HSP*_*ter*_ was assembled and inserted into the *Hind*III and *Nco*I sites of the pHEE401E plasmid (Wang et al. 2015), resulting in the *pHEE401E-UBQ10pro::dTvDAO-ploy A* (*dTvDAO-Cas9*) plasmid. The specific gRNAs targeting the three *Arabidopsis* endogenous genes were designed using CRISPR-GE (Xie et al. 2017). The polycistronic tRNA-gRNA repeats were synthesized through Golden Gate assembly, then amplified by PCR using primer pairs tRNA-F/HSP-R and inserted into the *I-Ceu*I site of the *dTvDAO-Cas9* plasmid to obtain the *pHEE401E-UBQ10pro::dTvDAO-ploy A-*(*tRNA-gRNA*)*s* (*dTvDAO-gRNAs-Cas9*) plasmid. The primers used for plasmid construction in this work are listed in Supplementary Table S1.

### D-serine tolerance assay in* E. coli*

The *E. coli* cells expressing *RgDAO*, *hDAO D31H*, *FvDAO*, *TvDAO*, *MmDAO*, or *CERK1* were cultured at 37 °C overnight. The cultures were then diluted to an OD_600_ of 0.5, 0.1, 0.02, and 0.005 before being applied onto LB medium containing 50 mg L^−1^ ampicillin, with or without 50 mM or 60 mM d-serine. The plates were incubated at 37 °C for approximately 20 h, and the tolerance phenotypes were recorded using a camera.

### Protoplast isolation and transfection

*Arabidopsis* protoplasts were extracted and transfected as previously described, using the leaves of 4-week-old plants (Yoo et al. 2007). The *dGFP* reporter was transiently expressed alone or co-expressed with *Cas9* and a cognate gRNA to the human target site in the *dGFP* reporter in *Arabidopsis* protoplasts. After overnight culture, the fluorescence was observed using a Leica DM8i C inverted microscope (Leica Biosystems, Germany).

### Generation of transgenic plants

The binary plasmids *TvDAO*, *dTvDAO-Cas9*, or *dTvDAO-gRNAs-Cas9* were introduced into *Agrobacterium tumefaciens* strain GV3101 by electroporation. The *Agrobacterium* cells carrying the appropriate binary plasmids were used to transform *Arabidopsis* using the widely used floral dip method (Clough and Bent 1998).

### Genomic DNA extraction

Crude genomic DNA (gDNA) extracts of *Arabidopsis* plants were obtained by homogenizing three leaves from a single plant in TKE buffer (100 mM Tris-HCl, pH 9.5, 1 M KCl, 10 mM EDTA), followed by incubation at 70 °C for 30 min. The tenfold diluted gDNA extract was used as a PCR template for genotyping.

### Genotyping of *Arabidopsis* plants

The Green Taq Mix (Vazyme, China) was used for genotyping. The PCR amplicons were separated by agarose gel electrophoresis. The gel strips containing PCR amplicons were excised and used for Sanger sequencing to validate the sequences. The primers used for genotyping PCR in this work are listed in Supplementary Table S1.

### Supplementary Information

Below is the link to the electronic supplementary material.

Supplementary file1 (DOCX 5829 KB)

## Data Availability

All data generated in this study are available in the paper or online Supplementary Information.

## References

[CR1] Clough SJ, Bent AF (1998). Floral dip: a simplified method for *Agrobacterium*-mediated transformation of *Arabidopsis thaliana*. Plant J.

[CR2] Cong L, Ran FA, Cox D, Lin S, Barretto R, Habib N, Hsu PD, Wu X, Jiang W, Marraffini LA, Zhang F (2013). Multiplex genome engineering using CRISPR/Cas systems. Science.

[CR3] Cullot G, Boutin J, Toutain J, Prat F, Pennamen P, Rooryck C, Teichmann M, Rousseau E, Lamrissi-Garcia I, Guyonnet-Duperat V, Bibeyran A, Lalanne M, Prouzet-Mauléon V, Turcq B, Ged C, Blouin JM, Richard E, Dabernat S, Moreau-Gaudry F, Bedel A (2019). CRISPR-Cas9 genome editing induces megabase-scale chromosomal truncations. Nat Commun.

[CR4] de Jong OG, MurphyDE MI, Willms E, Garcia-Guerra A, Gitz-Francois JJ, Lefferts J, Gupta D, Steenbeek SC, van Rheenen J, Andaloussi SE, Schiffelers RM, Wood MJA, Vader P (2020). A CRISPR-Cas9-based reporter system for single-cell detection of extracellular vesicle-mediated functional transfer of RNA. Nat Commun.

[CR5] Endo M, Iwakami S, Toki S (2021). Precision genome editing in plants via gene targeting and subsequent break-induced single-strand annealing. Plant Biotechnol J.

[CR6] Erikson O, Hertzberg M, Nasholm T (2004). A conditional marker gene allowing both positive and negative selection in plants. Nat Biotechnol.

[CR7] Feng Z, Mao Y, Xu N, Zhang B, Wei P, Yang DL, Wang Z, Zhang Z, Zheng R, Yang L, Zeng L, Liu X, Zhu JK (2014). Multigeneration analysis reveals the inheritance, specificity, and patterns of CRISPR/Cas-induced gene modifications in *Arabidopsis*. Proc Natl Acad Sci USA.

[CR8] Fiorenza MT, Bevilacqua A, Bevilacqua S, Mangia F (2001). Growing dictyate oocytes, but not early preimplantation embryos, of the mouse display high levels of DNA homologous recombination by single-strand annealing and lack DNA nonhomologous end joining. Dev Biol.

[CR9] Fu Y, Foden JA, Khayter C, Maeder ML, Reyon D, Joung JK, Sander JD (2013). High-frequency off-target mutagenesis induced by CRISPR-Cas nucleases in human cells. Nat Biotechnol.

[CR10] Gao X, Chen J, Dai X, Zhang D, Zhao Y (2016). An effective strategy for reliably isolating heritable and *Cas9*-free *Arabidopsis* mutants generated by CRISPR/Cas9-mediated genome editing. Plant Physiol.

[CR11] Gisby MF, Mudd EA, Day A (2012). Growth of transplastomic cells expressing D-amino acid oxidase in chloroplasts is tolerant to d-alanine and inhibited by d-valine. Plant Physiol.

[CR12] Gürel F, Zhang Y, Sretenovic S, Qi Y (2020) CRISPR-Cas nucleases and base editors for plant genome editing. aBIOTECH 1:74–87 10.1007/s42994-019-00010-0 PMC9584094 36305010

[CR13] He Y, Zhu M, Wang L, Wu J, Wang Q, Wang R, Zhao Y (2018). Programmed self-elimination of the CRISPR/Cas9 construct greatly accelerates the isolation of edited and transgene-free rice plants. Mol Plant.

[CR14] He Y, Zhao Y (2019) Technological breakthroughs in generating transgene-free and genetically stable CRISPR-edited plants. aBIOTECH 1:88–96 10.1007/s42994-019-00013-x PMC9584093 36305007

[CR15] Kirik V, Simon M, Wester K, Schiefelbein J, Hulskamp M (2004). *ENHANCER* of *TRY* and *CPC 2* (*ETC2*) reveals redundancy in the region-specific control of trichome development of *Arabidopsis*. Plant Mol Biol.

[CR16] Kong X, Pan W, Sun N, Zhang T, Liu L, Zhang H (2021). *GLABRA2*-based selection efficiently enriches Cas9-generated nonchimeric mutants in the T_1_ generation. Plant Physiol.

[CR17] Lao K, Xiao Y, Huang Q, Mo B, Dong X, Wang X (2023). Establishment of an efficient early flowering-assisted CRISPR/Cas9 gene-editing system in *Arabidopsis*. Plant Cell Rep.

[CR18] Lim S, Woo HJ, Lee SM, Jin YM, Cho H (2007). d*-amino acid oxidase *(*DAO*) gene as a novel selection marker for plant transformation. J Plant Biotechnol.

[CR19] Liu Y, Zeng J, Yuan C, Guo Y, Yu H, Li Y, Huang C (2019). Cas9-PF, an early flowering and visual selection marker system, enhances the frequency of editing event occurrence and expedites the isolation of genome-edited and transgene-free plants. Plant Biotechnol J.

[CR20] Lu HP, Liu SM, Xu SL, Chen WY, Zhou X, Tan YY, Huang JZ, Shu QY (2017). CRISPR-S: an active interference element for a rapid and inexpensive selection of genome-edited, transgene-free rice plants. Plant Biotechnol J.

[CR21] Oh JM, Myung K (2022). Crosstalk between different DNA repair pathways for DNA double strand break repairs. Mutat Res Genet Toxicol Environ Mutagen.

[CR22] Pilone MS (2000). d-amino acid oxidase: new findings. Cell Mol Life Sci.

[CR23] Pollegioni L, Piubelli L, Sacchi S, Pilone MS, Molla G (2007). Physiological functions of d-amino acid oxidases: from yeast to humans. Cell Mol Life Sci.

[CR24] Pollegioni L, Sacchi S, Murtas G (2018). Human d-amino acid oxidase: structure, function, and regulation. Front Mol Biosci.

[CR25] Rinne J, Witte CP, Herde M (2021). Loss of *MAR1* function is a marker for co-selection of CRISPR-induced mutations in plants. Front Genome Ed.

[CR26] Stinson BM, Loparo JJ (2021). Repair of DNA double-strand breaks by the nonhomologous end joining pathway. Annu Rev Biochem.

[CR27] Tang X, Ren Q, Yang L, Bao Y, Zhong Z, He Y, Liu S, Qi C, Liu B, Wang Y, Sretenovic S, Zhang Y, Zheng X, Zhang T, Qi Y, Zhang Y (2019). Single transcript unit CRISPR 2.0 systems for robust Cas9 and Cas12a mediated plant genome editing. Plant Biotechnol J.

[CR28] Tian Y, Zhong D, Li X, Shen R, Han H, Dai Y, Yao Q, Zhang X, Deng Q, Cao X, Zhu JK, Lu Y (2023). High-throughput genome editing in rice with a virus-based surrogate system. J Integr Plant Biol.

[CR29] Tsutsui H, Higashiyama T (2017). pKAMA-ITACHI vctors for highly efficient CRISPR/Cas9-mediated gene knockout in *Arabidopsis thaliana*. Plant Cell Physiol.

[CR30] Vu TV, Das S, Nguyen CC, Kim J, Kim JY (2022). Single-strand annealing: molecular mechanisms and potential applications in CRISPR-Cas-based precision genome editing. Biotechnol J.

[CR32] Wang J, Chen H (2020). A novel CRISPR/Cas9 system for efficiently generating *Cas9*-free multiplex mutants in *Arabidopsis*. aBIOTECH.

[CR31] Wang ZP, Xing HL, Dong L, Zhang HY, Han CY, Wang XC, Chen QJ (2015). Egg cell-specific promoter-controlled CRISPR/Cas9 efficiently generates homozygous mutants for multiple target genes in *Arabidopsis* in a single generation. Genome Biol.

[CR33] Wolabu TW, Park JJ, Chen M, Cong L, Ge Y, Jiang Q, Debnath S, Li G, Wen J, Wang Z (2020). Improving the genome editing efficiency of CRISPR/Cas9 in *Arabidopsis* and *Medicago truncatula*. Planta.

[CR34] Xie K, Minkenberg B, Yang Y (2015). Boosting CRISPR/Cas9 multiplex editing capability with the endogenous tRNA-processing system. Proc Natl Acad Sci USA.

[CR35] Xie X, Ma X, Zhu Q, Zeng D, Li G, Liu YG (2017). CRISPR-GE: a convenient software toolkit for CRISPR-based genome editing. Mol Plant.

[CR36] Xu W, Yang Y, Liu Y, Kang G, Wang F, Li L, Lv X, Zhao S, Yuan S, Song J, Wu Y, Feng F, He X, Zhang C, Song W, Zhao J, Yang J (2020). Discriminated sgRNAs-based surrogate system greatly enhances the screening efficiency of plant base-edited cells. Mol Plant.

[CR37] Xu R, Li J, Liu X, Shan T, Qin R, Wei P (2020). Development of plant prime-editing systems for precise genome editing. Plant Commun.

[CR38] Yoo SD, Cho YH, Sheen J (2007). *Arabidopsis* mesophyll protoplasts: a versatile cell system for transient gene expression analysis. Nat Protoc.

[CR39] Zhang Q, Xing HL, Wang ZP, Zhang HY, Yang F, Wang XC, Chen QJ (2018). Potential high-frequency off-target mutagenesis induced by CRISPR/Cas9 in *Arabidopsis* and its prevention. Plant Mol Biol.

