# Supplementary material for: A dual-function selection system enables positive selection of multigene CRISPR mutants and negative selection of Cas9-free progeny in Arabidopsis
Source: aBIOTECH. 2024 Jan 22;5(2):140–50. doi: 10.1007/s42994-023-00132-6 (PMC11224197; doi:10.1007/s42994-023-00132-6)
Supplement: Supplementary file 1 — Supplementary file1 (DOCX 5829 KB) [file 42994_2023_132_MOESM1_ESM.docx]

**Supplementary Information**

**A dual-function selection system enables positive selection of multigene CRISPR mutants and negative selection of *Cas9*-free progeny in *Arabidopsis***

Feng-Zhu Wang^1,2,*^, Ying Bao^1,2^, Zhenxiang Li^1^, Xiangyu Xiong^1^, Jian-Feng Li^1,*^

^1^State Key Laboratory of Biocontrol, Guangdong Provincial Key Laboratory of Plant Resources, School of Life Sciences, Sun Yat-sen University, Guangzhou 510275, China

^2^These authors contributed equally to this work

^*^Correspondence: [wangfzh5@mail.sysu.edu.cn](mailto:wangfzh5@mail.sysu.edu.cn) (F.-Z. Wang), [lijfeng3@mail.sysu.edu.cn](mailto:lijfeng3@mail.sysu.edu.cn) (J.-F. Li)


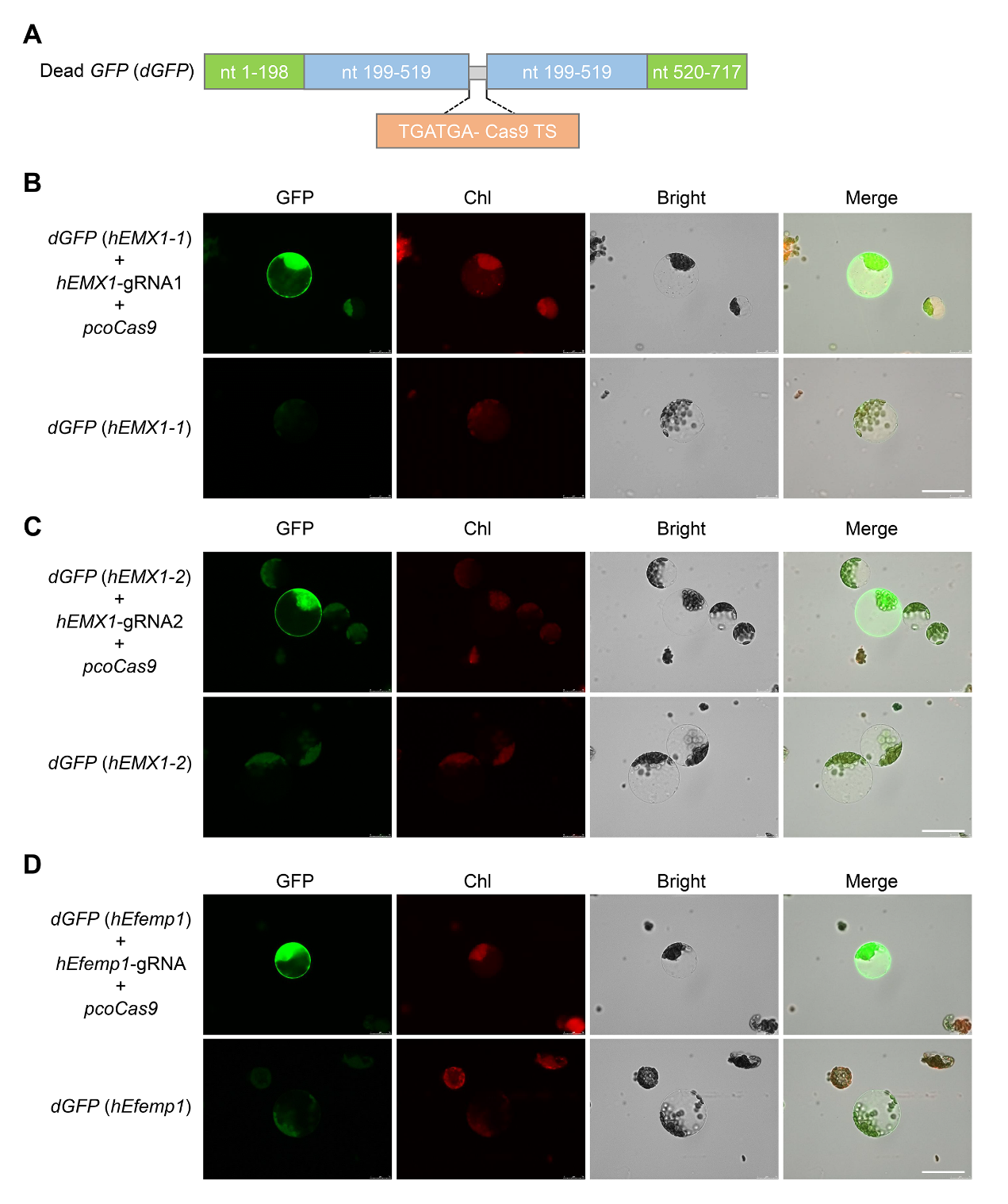


**Fig. S1 | CRISPR-induced SSA repair can rescue a dead *GFP* reporter in *Arabidopsis* protoplasts.**

**A** Diagram of the dead *GFP* (*dGFP*) reporter. The most 5’ (1-198) and 3’ segments (520-717) of *GFP* were colored in green. The middle segment (199-519) of *GFP* was colored in blue and the two copies of it were spacered by two consecutive stop codons and a Cas9 target site from the human genome. nt, nucleotides. **B-D** CRISPR-induced SSA repair successfully recovers an active *GFP*. In **B**, the human *hEXM1-1* site was used in the *dGFP* reporter and its cognate gRNA, *hEXM1*-gRNA1, was expressed. In **C**, the human *hEXM1-2* site was used in the *dGFP* reporter and its cognate gRNA, *hEXM1*-gRNA2, was expressed. In **C**, the human *hEfemp1* site was used in the *dGFP* reporter and its cognate gRNA, *hEfemp1*-gRNA1, was expressed. Chl, chlorophyl autofluorescence. Scale bar = 50 μm.


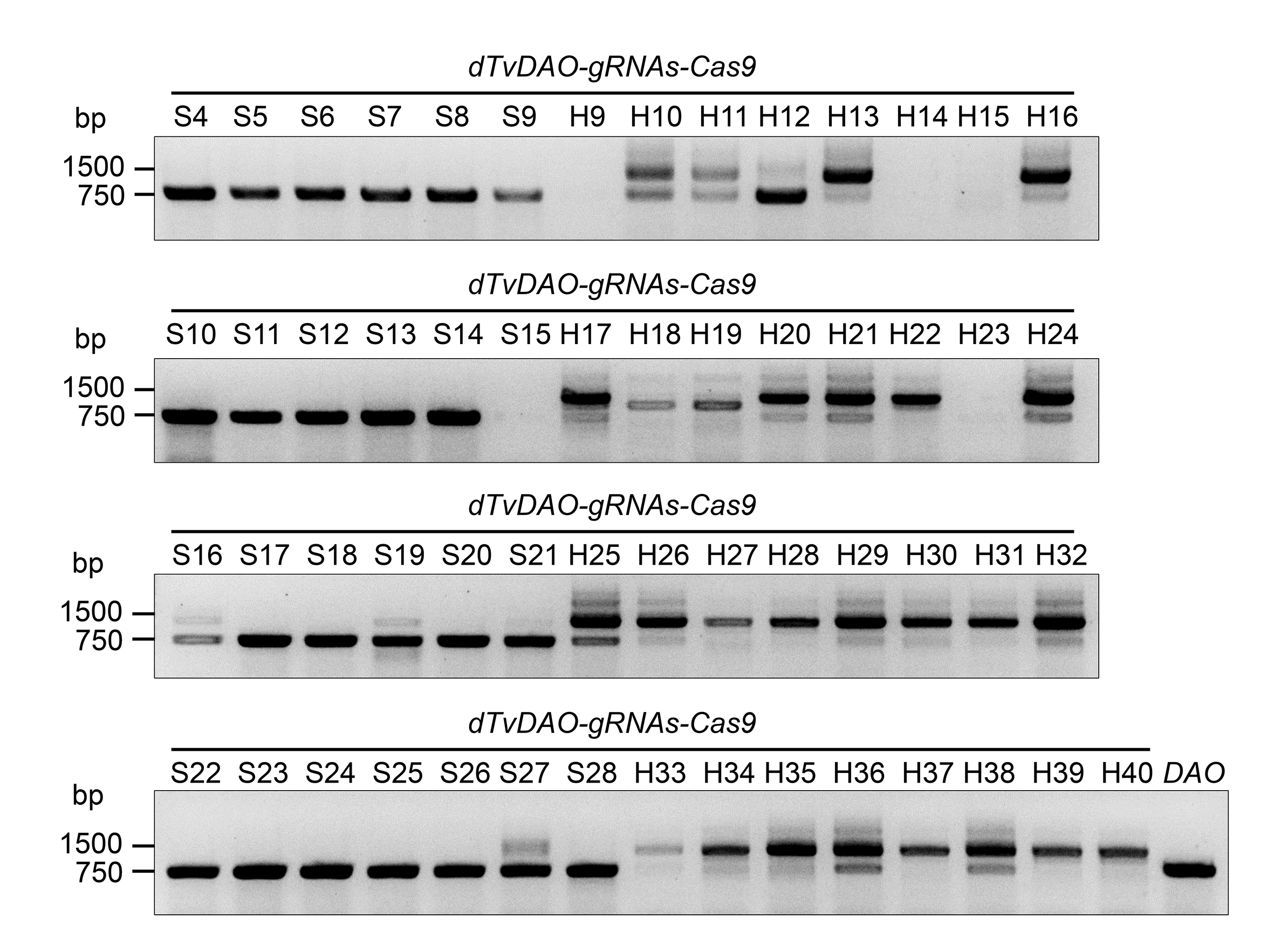


**Fig. S2 | PCR-based genotyping confirms successful SSA repair of *dTvDAO* in D-serine-resistant transgenic T_1_ plants.** S, D-serine selection. H, hygromycin selection. Transgenic *TvDAO* #2 plant was used as a positive control (*DAO*).

**
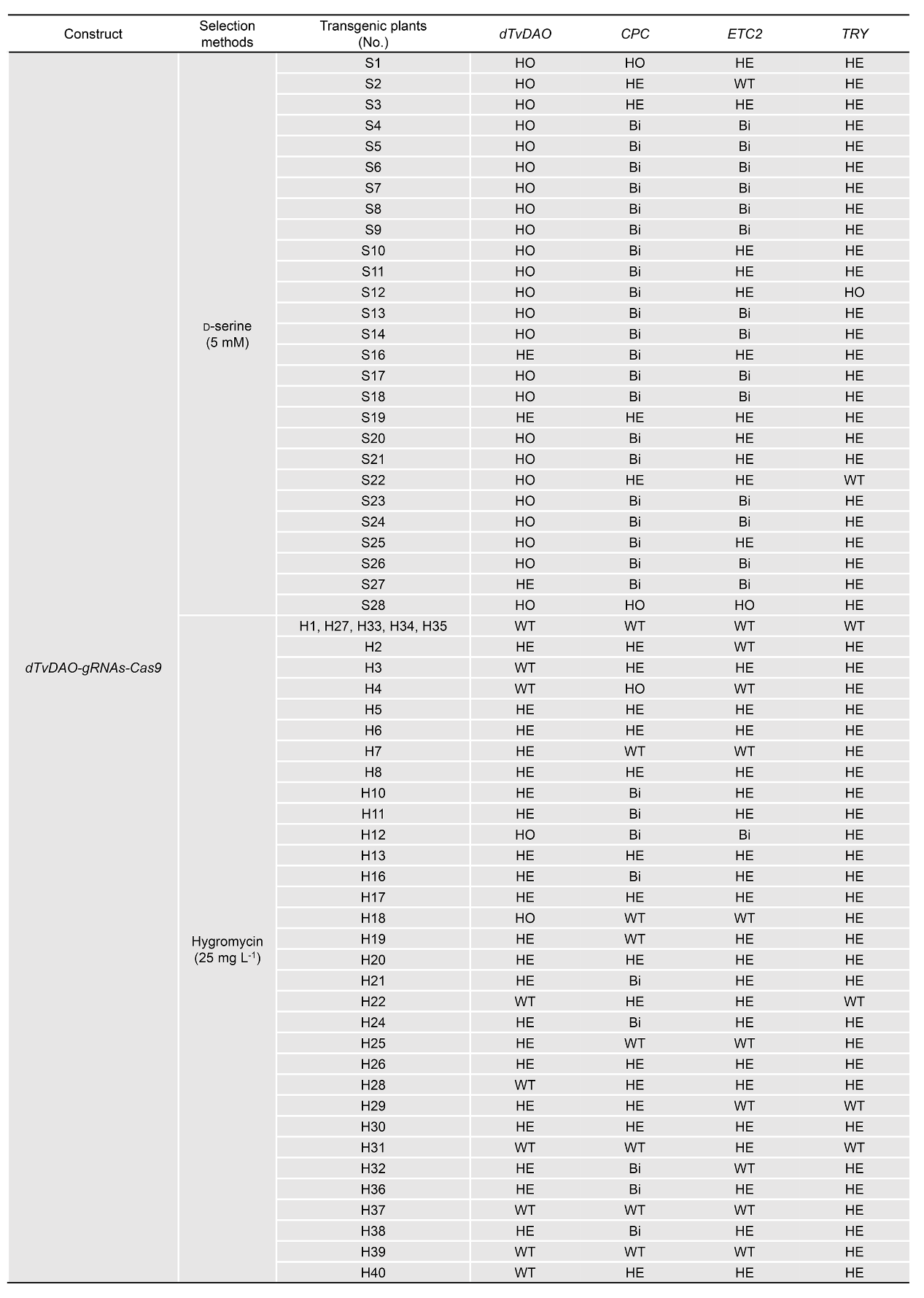
**

**Fig. S3 | Detailed mutation information for individual transgenic T_1_ plants selected by D-serine or hygromycin.**

HO, HE, Bi and WT represent homozygote, heterozygote, bi-allele and wild type, respectively. Note that S15, H9, H14, H15 and H23 were excluded for this analysis as they were non-transgenic (Fig. S2).


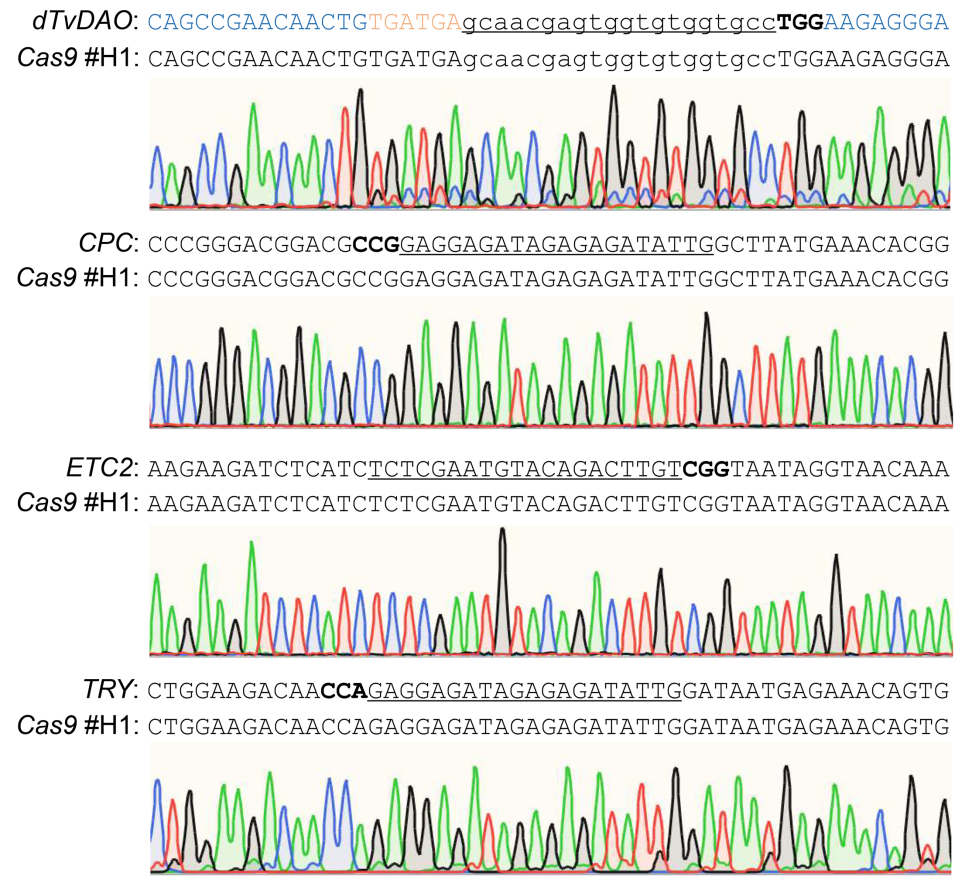


**Fig. S4 | Sanger sequencing reveals no mutation at the four target sites in the *Cas9* #H1 plant selected by hygromycin.**

Blue letters in *dTvDAO* represent the C-terminal coding sequence of LH or N-terminal coding sequence of RH. Orange letters represent double stop codons. The target site of *hEfemp1*-gRNA is underlined and in lowercase. Black bold letters mark PAMs and target sequences of gRNAs are underlined.

**Supplementary** **Table S1. The list of primers used in this article**

| **Primer name** | **Sequence (5'-3')** | **Usage** |
| --- | --- | --- |
| BamHI-GFP-F | CGTGGATCCATGGTGAGCAAGGGCGAG | For construction of the plasmids *HBT-35SPPDK-dGFP(TS)-2×HA* |
| GFP-hEMX1-1-R | TTCTTCTTCTGCTCGGACTCTCATCACTCGATGTTGTGGCGGATC |  |
| hEMX1-1-GFP-F | GAGTCCGAGCAGAAGAAGAAGGGTACGGCGTGCAGTGCTTCA |  |
| GFP-hEMX1-2-R | TAGTCATTGGAGGTGACATCTCATCACTCGATGTTGTGGCGGATC |  |
| hEMX1-2-GFP-F | GATGTCACCTCCAATGACTAGGGTACGGCGTGCAGTGCTTCA |  |
| GFP-hEfemp1-R | ggcaccacaccactcgttgcTCATCACTCGATGTTGTGGCGGATC |  |
| hEfemp1-GFP-F | gcaacgagtggtgtggtgccTGGTACGGCGTGCAGTGCTTCA |  |
| GFP-HA-R | CGTCGTATGGGTAAGGCCTCTTG |  |
| hEMX1-gRNA1-F | ATTGgagtccgagcagaagaagaa | For construction of the plasmid *pUC119-AtU6-26::hEMX1 gRNA1* |
| hEMX1-gRNA1-R | AAACttcttcttctgctcggactc |  |
| hEMX1-gRNA2-F | ATTGctccccattggcctgcttcg | For construction of the plasmid *pUC119-AtU6-26::hEMX1 gRNA2* |
| hEMX1-gRNA2-R | AAACcgaagcaggccaatggggag |  |
| hEfemp1-gRNA-F | ATTGgcaacgagtggtgtggtgcc | For construction of the plasmid *pUC119-AtU6-26::hEfemp1 gRNA* |
| hEfemp1-gRNA-R | AAACggcaccacaccactcgttgc |  |
| BamHI-UBQ10p-F | aggtagcgaaggatccTCATTCTTGTTACATTGTTATTAATG | For construction of the plasmid *pYL-UBQ10pro::TvDAO-2HA-polyA-HSP_ter_* |
| UBQ10-R | ATCTGTTAATCAGAAAAACTCAGATTAATC |  |
| UBQ-TvD-F | TTCTGATTAACAGATATGGCCAAGATCGTGGTCAT |  |
| TvDAO-HA-R | CGTATGGGTAAGGCCTGAGATTCGGCCTG |  |
| TvDAO-HA-F | AGGCCTTACCCATACGACG |  |
| HA-polyA-R | CTTAGGACCGTTATAGTTAttttttttttttttttttttttttttttttttttttttttttttttttttCTGCAGTCAAGCGTAGTCTG |  |
| ICeuI-HSP-F | TAACTATAACGGTCCTAAGGTAGCGAATATGAAGATGAAGATGAAATATTTGGTG |  |
| StuI-HSP-R | cattcgccattcaggcctCTTATCTTTAATCATATTCCATAGTCCATACCATAG |  |
| HindIII-UBQ10-F | ggccagtgccaagcttTCATTCTTGTTACATTGTTATTAATGAAAAAATATTATTG | For construction of the plasmid *pHEE401E-UBQ10pro::dTvDAO-polyA* |
| TvD-hEfemp1-R | caccacaccactcgttgcTCATCACAGTTGTTCGGCTGGAAGC |  |
| HEfemp1-TvD-F | caacgagtggtgtggtgccTGGAAGAGGGACCTCCCGAAGC |  |
| NcoI-HSP-R | actagtataaccatggCTTATCTTTAATCATATTCCATAGTCCATACCATAGC |  |
| polyA-tRNA-F | aTAACTATAACGGTCAACAAAGCACCAGTGGTCTAGTGG | For construction of the plasmid *pHEE401E-UBQ10pro::dTvDAO- ployA-(tRNA-gRNA)s* |
| BasI-hEfemp1 gRNA-R | cgaGGTCTCAaccactcgttgcTGCACCAGCCGGGAATC |  |
| BasI-hEfemp1 gRNA-F | cgaGGTCTCCtggtgtggtgccGTTTTAGAGCTAGAAATAGCAAG |  |
| BasI-TRY CPC gRNA-F | cgaGGTCTCCctctatctcctcGTTTTAGAGCTAGAAATAGCAAG |  |
| BasI-TRY CPC gRNA-R | cgaGGTCTCAagagagatattgTGCACCAGCCGGGAATC |  |
| BasI-ETC2 gRNA-F | cgaGGTCTCCgtacagacttgtGTTTTAGAGCTAGAAATAGCAAG |  |
| BasI-ETC2 gRNA-R | cgaGGTCTCAgtacattcgagaTGCACCAGCCGGGAATC |  |
| tRNA-HSP-R | TTCATATTCGCTACCTGCACCAGCCGGGAATCG |  |
| tRNA-F | aTAACTATAACGGTCAACAAAGC |  |
| HSP-R | TTCATATTCGCTACCTGCAC |  |
| ETC2 id-F | CCAGTAGTTATGGATAATACCAACCG | For genotyping of *ETC2*, *CPC*, *TRY* |
| ETC2 id-R | GATTTGTTACTCTCGCCATGTG |  |
| CPC id-F | CCGAGCTGTCAATGACTGTG |  |
| CPC id-R | CATGTGTCGATGGAGGCTGG |  |
| TRY id-F | GTAGGGGAAGCACATGGTGTC |  |
| TRY id-R | CCTAACCGCATGGATTAAAGTTG |  |
| AtACTIN2-F | GGTTGGGATGAACCAGAAGG | For amplify gene *AtACTIN2* |
| AtACTIN2-R | CCATCTCCTGCTCGTAGTCAAC |  |
| UBQ10-F | CTGGGTTTGATCGTTAGATATCATC | For Sanger sequencing of DNA constructs or PCR amplicons |
| UBQ10-seq-F2 | CTCGATTAGGGTTTCATAAATATC |  |
| UBQ10-seq-F3 | TCTGTGTTCCTTATTCTCTC |  |
| TvDAO-F | GAGGTGACCATCGTCTCCG |  |
| TvDAO-F2 | GACGCGGTCTCCTACCCG |  |
| TvDAO-seq-F2 | GAGGGAGTGCGTGGGC |  |
| TvDAO-seq-R | CAAGATCCTGTGGGTGAGG |  |
| hEfemp1-seq-F | CGAGTGGTGTGGTGCC |  |
| HSP-seq-R | TCATAACACAACAAGCCAAG |  |
| pHEE-Cas9-R | CGTCAACCTTCGCCATCTC |  |
| TRY-seq-F | GTATATAAATGCTTGGCTGGCTC |  |

**Supplementary Sequences**

**>**The coding sequence of *TvDAO*

ATGGCCAAGATCGTGGTCATCGGCGCCGGCGTCGCGGGACTTACCACCGCTCTCCAGCTCCTGAGGAAGGGCCACGAGGTGACCATCGTCTCCGAGTTCACCCCAGGTGACCTCAGCATCGGCTACACCTCCCCGTGGGCGGGCGCTAACTGGCTGACCTTCTACGATGGCGGCAAGCTGGCCGACTACGACGCGGTCTCCTACCCGATCCTCCGCGAGCTGGCGCGCAGCTCTCCGGAAGCTGGCATCCGCCTGATCAGCCAGCGCTCCCACGTGCTCAAGAGGGACCTCCCGAAGCTCGAAGTGGCCATGTCCGCCATCTGCCAGAGGAACCCGTGGTTCAAGAATACCGTGGATAGCTTCGAGATCATCGAGGATCGCTCCAGGATCGTCCACGACGATGTGGCGTACCTCGTGGAGTTCCGCTCCGTGTGCATCCACACCGGCGTCTACCTGAATTGGCTGATGTCCCAGTGCCTCAGCCTCGGCGCGACCGTGGTGAAGAGGAGGGTGAATCACATCAAGGATGCCAACCTGCTCCACTCCTCCGGCAGCAGGCCGGATGTGATCGTGAATTGCAGCGGCCTGTTCGCCCGCTTCCTCGGCGGAGTGGAGGACAAGAAGATGTACCCGATCCGCGGCCAGGTGGTCCTGGTGAGGAATAGCCTCCCGTTCATGGCCTCCTTCTCCAGCACCCCGGAGAAGGAGAACGAGGACGAGGCCCTGTACATCATGACCAGGTTCGACGGCACCAGCATCATCGGCGGCTGCTTCCAGCCGAACAACTGGTCCTCCGAGCCGGACCCTAGCCTCACCCACAGGATCTTGTCCCGCGCCCTGGACAGGTTCCCGGAGCTGACCAAGGATGGCCCGCTCGATATTGTGAGGGAGTGCGTGGGCCATAGGCCGGGCAGGGAGGGAGGACCAAGGGTGGAGCTGGAGAAGATCCCAGGTGTGGGCTTCGTCGTCCATAATTACGGCGCCGCCGGCGCCGGCTACCAGTCCTCCTATGGCATGGCCGACGAGGCCGTGTCCTACGTGGAGAGGGCGCTCACCAGGCCGAATCTC

**>**The coding sequence of *RgDAO*

ATGCACAGCCAGAAGAGGGTGGTGGTCCTGGGCTCCGGCGTGATCGGCCTGTCCAGCGCTCTCATCCTGGCGAGGAAGGGCTACTCCGTGCATATCGTCGCCCGCGATCTGCCGGAGGACGTGTCCAGCCAGACCTTCGCCTCCCCGTGGGCGGGAGCTAATTGGACCCCGTTCATGAGCCTCACCGATGGCCCGCGCCAGGCGAAGTGGGAGGAGCTTACCTTCAAGAAGTGGGTGGAGCTGGTGCCGACCGGCCAGGTCATGTGGCTCAAGGGCACCAGGAGGTTCGCCCAGAATGAGGATGGCCTCCTGGGCCACTGGTACAAGGACATCACCCCGAATTACCGCCCGCTGCCGAGCAGCGAGTGCCCACCAAATTCCATCGGCGTGACCTACGATACCCTGTCCGTGCACGCCCCGAAGTACTGCCAGTACCTCGCGAGGGGCCTGCAAAAGCTCGGCGCCACCTTCGAGCGCCGCACCGTTACCTCCGTCGAGCAGGCGTTCGAGGGCGTGGACCTCGTGGTCAATGCCACCGGCCTCGGCGCGAAGTCCATCGCTGGCATCGATGATCAGGCGGCGGAGCCGATCCGCGGCCAAACAGTCCTCGTGAAGTCCGCGTGCAAGCGCTGCACCATGGACTCCTCCGATCCGTCCTCCCCGGCGTACATCATCCCGAGGCCGGGCGGAGAGGTCATTTGCGGCGGCACCTACGGCGTCGGCGACTGGGATCTCAGCGTCAACCCGGAGACCGTCCAGAGGATCTTGAAGCATTGCCTCCGCCTGGACCCTAGCATCTCCTCCGACGGCACCATCGAGGGCATCGAGGTGCTCCGCCATAACGTGGGCCTGCGCCCGGCTAGGAGGGGAGGACCAAGGGTCGAGGCGGAGAGGCTGGTGCTGCCGCTCGACAGGAGCAAGTCCCCGCTGTCCCTCGGCAAGGGCACCACCAGGGCGGCTAAGGAGAAGGAGGTGACCCTCGTGCACGCGTACGGCTTCTCCTCCGCGGGCTACCAGCAGAGCTGGGGCGCTGCTGAGGACGTGGCGCTCCTGGTGGAGGAGGCGTTCCAGAGGTACCACGGCGCGGCCAGGGAGAGCAAGCTC

**>**The coding sequence of *hDAO D31H*

ATGCGCGTCGTGGTCATCGGCGCGGGCGTGATCGGCCTGAGCACCGCTCTGTGCATCCACGAGAGGTACCACAGCGTCCTGCAACCGCTGCACATCAAGGTCTACGCGGACAGGTTCACCCCGCTGACCACCACCGATGTGGCCGCGGGACTCTGGCAGCCGTACCTGTCCGATCCGAACAACCCGCAGGAGGCCGATTGGAGCCAGCAGACCTTCGACTACCTGCTGTCCCATGTCCATAGCCCGAACGCCGAGAACCTGGGCCTGTTCCTGATCAGCGGCTACAACCTGTTCCACGAGGCCATCCCGGACCCGAGCTGGAAGGACACCGTCCTGGGCTTCCGCAAGCTCACCCCGCGCGAGCTGGATATGTTCCCGGACTACGGCTACGGCTGGTTCCACACCTCCCTGATCCTGGAGGGCAAGAATTACCTGCAATGGCTCACCGAGCGCCTCACCGAGCGGGGAGTGAAGTTCTTCCAGCGCAAGGTCGAGAGCTTCGAGGAGGTGGCCCGCGAGGGCGCTGATGTGATCGTCAACTGCACCGGCGTCTGGGCGGGCGCTCTCCAAAGGGACCCGCTCCTGCAACCGGGCAGGGGACAGATCATGAAGGTGGATGCCCCGTGGATGAAGCATTTCATCCTCACCCATGATCCGGAGCGCGGCATCTACAACAGCCCGTACATCATCCCAGGTACCCAGACCGTCACCCTCGGCGGCATCTTCCAGCTCGGCAACTGGAGCGAGCTGAATAACATCCAGGACCACAATACCATCTGGGAGGGCTGCTGCCGGCTCGAACCGACCCTCAAGAACGCCAGGATCATCGGCGAGAGGACCGGCTTCAGGCCGGTGAGGCCGCAGATCCGCCTCGAAAGGGAGCAGCTCAGGACCGGCCCGTCCAATACCGAGGTCATCCATAATTACGGCCACGGCGGCTACGGCCTGACCATCCACTGGGGCTGCGCCCTCGAAGCGGCGAAGCTCTTCGGCCGCATCCTCGAAGAGAAGAAGCTCTCCCGCATGCCGCCGTCCCATCTC

**>**The coding sequence of *FvDAO*

ATGTCCAATACCATCGTGGTCGTGGGCGCCGGCGTCATCGGCCTTACCTCCGCTCTCCTGCTCAGCAAGAACAAGGGCAACAAGATCACCGTCGTGGCGAAGCATATGCCGGGCGACTACGACGTGGAGTACGCGAGCCCGTTCGCCGGCGCTAATCACAGCCCGATGGCGACCGAGGAGTCCTCCGAGTGGGAGCGCCGCACCTGGTACGAGTTCAAGCGCCTCGTCGAGGAGGTGCCGGAGGCGGGAGTCCATTTCCAGAAGTCCAGGATTCAACGCAGGAACGTGGACACCGAGAAGGCCCAGAGGTCCGGCTTCCCGGATGCCCTGTTCAGCAAGGAGCCGTGGTTCAAGAATATGTTCGAGGACTTCAGGGAGCAGCACCCGTCCGAGGTCATCCCAGGTTACGACAGCGGCTGCGAGTTCACCAGCGTCTGCATCAATACCGCCATCTACCTGCCGTGGCTGCTCGGCCAGTGCATCAAGAACGGCGTCATCGTCAAGAGGGCCATCCTGAATGACATCTCCGAGGCCAAGAAGCTGAGCCACGCCGGCAAGACCCCGAACATCATCGTCAACGCCACCGGCCTCGGCAGCTACAAGCTCGGCGGCGTCGAGGACAAGACCATGGCCCCGGCCCGCGGACAGATCGTCGTGGTTAGGAATGAGTCCTCCCCGATGCTGCTGACCTCCGGCGTGGAGGACGGCGGAGCTGACGTGATGTACCTGATGCAGCGCGCGGCCGGCGGCGGAACCATTCTTGGAGGCACCTACGACGTCGGCAACTGGGAGTCCCAGCCGGACCCGAATATCGCCAACAGGATCATGCAGAGGATCGTCGAGGTCAGGCCGGAGATCGCGAATGGCAAGGGCGTGAAGGGCCTGTCCGTCATCAGGCATGCCGTGGGCATGAGGCCGTGGCGCAAGGACGGCGTCAGGATCGAGGAGGAGAAGCTCGACGATGAGACCTGGATCGTGCACAACTACGGCCATAGCGGCTGGGGCTACCAGGGCTCCTACGGCTGCGCGGAGAACGTGGTCCAGCTCGTCGACAAGGTGGGCAAGGCGGCGAAGTCCAAGCTG

**>**The coding sequence of *MmDAO*

ATGCGCGTGGCCGTCATCGGCGCCGGAGTGATCGGCCTCAGCACCGCTCTCTGCATCCATGAGCGCTACCACCCGACCCAGCCGCTCCACATGAAGATCTACGCCGACCGCTTCACCCCGTTCACCACCAGCGATGTCGCGGCCGGCCTCTGGCAGCCATACCTGTCCGATCCGTCCAATCCGCAGGAGGCCGAGTGGAGCCAGCAGACCTTCGATTACCTCCTGTCCTGCCTGCACAGCCCGAATGCCGAGAAGATGGGCCTGGCGCTGATCAGCGGCTACAACCTCTTCAGGGATGAGGTGCCGGACCCTTTCTGGAAGAACGCCGTGCTCGGCTTCCGCAAGCTCACCCCGAGCGAGATGGATCTGTTCCCGGATTACGGCTACGGCTGGTTCAACACCAGCCTCCTCCTGGAGGGCAAGAGCTACCTCCCGTGGCTGACCGAGAGGCTCACCGAGAGGGGCGTGAAGCTGATCCACCGCAAGGTGGAGAGCCTCGAAGAGGTGGCGAGGGGCGTCGACGTGATCATCAACTGCACCGGCGTGTGGGCGGGCGCCCTTCAAGCTGATGCCAGCCTGCAACCGGGCCGCGGACAGATCATCCAGGTGGAGGCCCCGTGGATCAAGCATTTCATCCTCACCCATGATCCGTCCCTGGGCATCTACAACTCCCCGTACATCATCCCAGGTAGCAAGACCGTCACCCTGGGCGGCATCTTCCAGCTCGGCAACTGGTCCGGCCTGAATAGCGTCCGCGACCACAACACCATCTGGAAGAGCTGCTGCAAGCTCGAACCGACCCTCAAGAATGCCCGCATCGTCGGCGAGCTGACCGGCTTCAGGCCGGTGAGGCCGCAGGTGAGGCTCGAACGCGAGTGGCTGCGCCATGGCTCCAGCTCCGCGGAGGTCATCCACAACTACGGCCATGGCGGCTACGGCCTGACCATCCACTGGGGCTGCGCGATGGAGGCCGCCAATCTCTTCGGCAAGATCCTCGAAGAGAAGAAGCTCTCCCGCCTCCCGCCGAGCCACCTC

**>**The coding sequence of *dTvDAO*

[the coding sequence for LH and RH of *TvDAO* are in dark blue; the coding sequence for N terminal and C terminal of *TvDAO* are in green; the double stop codons are in orange; target sequence of *hEfemp1*-gRNA is underlined and PAM is in bold]

ATGGCCAAGATCGTGGTCATCGGCGCCGGCGTCGCGGGACTTACCACCGCTCTCCAGCTCCTGAGGAAGGGCCACGAGGTGACCATCGTCTCCGAGTTCACCCCAGGTGACCTCAGCATCGGCTACACCTCCCCGTGGGCGGGCGCTAACTGGCTGACCTTCTACGATGGCGGCAAGCTGGCCGACTACGACGCGGTCTCCTACCCGATCCTCCGCGAGCTGGCGCGCAGCTCTCCGGAAGCTGGCATCCGCCTGATCAGCCAGCGCTCCCACGTGCTCAAGAGGGACCTCCCGAAGCTCGAAGTGGCCATGTCCGCCATCTGCCAGAGGAACCCGTGGTTCAAGAATACCGTGGATAGCTTCGAGATCATCGAGGATCGCTCCAGGATCGTCCACGACGATGTGGCGTACCTCGTGGAGTTCCGCTCCGTGTGCATCCACACCGGCGTCTACCTGAATTGGCTGATGTCCCAGTGCCTCAGCCTCGGCGCGACCGTGGTGAAGAGGAGGGTGAATCACATCAAGGATGCCAACCTGCTCCACTCCTCCGGCAGCAGGCCGGATGTGATCGTGAATTGCAGCGGCCTGTTCGCCCGCTTCCTCGGCGGAGTGGAGGACAAGAAGATGTACCCGATCCGCGGCCAGGTGGTCCTGGTGAGGAATAGCCTCCCGTTCATGGCCTCCTTCTCCAGCACCCCGGAGAAGGAGAACGAGGACGAGGCCCTGTACATCATGACCAGGTTCGACGGCACCAGCATCATCGGCGGCTGCTTCCAGCCGAACAACTGTGATGAgcaacgagtggtgtggtgcc**TGG**AAGAGGGACCTCCCGAAGCTCGAAGTGGCCATGTCCGCCATCTGCCAGAGGAACCCGTGGTTCAAGAATACCGTGGATAGCTTCGAGATCATCGAGGATCGCTCCAGGATCGTCCACGACGATGTGGCGTACCTCGTGGAGTTCCGCTCCGTGTGCATCCACACCGGCGTCTACCTGAATTGGCTGATGTCCCAGTGCCTCAGCCTCGGCGCGACCGTGGTGAAGAGGAGGGTGAATCACATCAAGGATGCCAACCTGCTCCACTCCTCCGGCAGCAGGCCGGATGTGATCGTGAATTGCAGCGGCCTGTTCGCCCGCTTCCTCGGCGGAGTGGAGGACAAGAAGATGTACCCGATCCGCGGCCAGGTGGTCCTGGTGAGGAATAGCCTCCCGTTCATGGCCTCCTTCTCCAGCACCCCGGAGAAGGAGAACGAGGACGAGGCCCTGTACATCATGACCAGGTTCGACGGCACCAGCATCATCGGCGGCTGCTTCCAGCCGAACAACTGGTCCTCCGAGCCGGACCCTAGCCTCACCCACAGGATCTTGTCCCGCGCCCTGGACAGGTTCCCGGAGCTGACCAAGGATGGCCCGCTCGATATTGTGAGGGAGTGCGTGGGCCATAGGCCGGGCAGGGAGGGAGGACCAAGGGTGGAGCTGGAGAAGATCCCAGGTGTGGGCTTCGTCGTCCATAATTACGGCGCCGCCGGCGCCGGCTACCAGTCCTCCTATGGCATGGCCGACGAGGCCGTGTCCTACGTGGAGAGGGCGCTCACCAGGCCGAATCTC
